# An Artificial Synapse Based on CsPbI3 Thin Film

**DOI:** 10.3390/mi13020284

**Published:** 2022-02-10

**Authors:** Jia-Ying Chen, Xin-Gui Tang, Qiu-Xiang Liu, Yan-Ping Jiang, Wen-Min Zhong, Fang Luo

**Affiliations:** Guangzhou Higher Education Mega Center, School of Physics and Optoelectric Engineering, Guangdong University of Technology, Guangzhou 510006, China; 2112015044@mail2.gdut.edu.cn (J.-Y.C.); liuqx@gdut.edu.cn (Q.-X.L.); ypjiang@gdut.edu.cn (Y.-P.J.); zhongwen_min@163.com (W.-M.Z.); luofang_grsy9950@sina.com (F.L.)

**Keywords:** artificial synapse, long-term synaptic plasticity, short-term synaptic plasticity, halide perovskite

## Abstract

With the data explosion in the intelligent era; the traditional von Neumann computing system is facing great challenges of storage and computing speed. Compared to the neural computing system, the traditional computing system has higher consumption and slower speed. However; the feature size of the chip is limited due to the end of Moore’s Law. An artificial synapse based on halide perovskite CsPbI_3_ was fabricated to address these problems. The CsPbI_3_ thin film was obtained by a one-step spin-coating method, and the artificial synapse with the structure of Au/CsPbI_3_/ITO exhibited learning and memory behavior similar to biological neurons. In addition, the synaptic plasticity was proven, including short-term synaptic plasticity (STSP) and long-term synaptic plasticity (LTSP). We also discuss the possibility of forming long-term memory in the device through changing input signals.

## 1. Introduction

Integrated circuits have faced challenges. The large amounts of data limit the speed of calculation. The requirement of high-speed calculations causes large consumption. However, the end of Moore’s Law means that the pursuit of high integration is no longer realistic. Therefore, it is necessary to design a high-speed, low-consumption computing system. In the brain, a nerve pulse arrives at each synapse about 10 times/s, on average. There are about 10^15^ synapses, so the brain accomplishes almost 10^15^ complex operations/s, with a cost of no more than 10^6^ J each time [1,2]. As a new idea for solving problems, artificial synapses have attracted huge interest and have been widely studied.

Halide perovskites are advanced in artificial synapses, for which various defects in halide perovskites result in tunable charge capture capability. Tunable charge capture capability means alterable charge conductance and retention. In addition, halide perovskites have low halide ion migration activation energy, which is a convenient and optimized ionic semiconductor with excellent optical properties, providing conditions for the optical signal to become an effective modulation method for low-energy artificial synaptic devices [3]. Xu et al. reported an artificial synapse made from a bromine-containing organic halide perovskite (OHP), MAPbBr_3_. This was the first time that OHP was applied to an artificial synapse [4]. Halide perovskite is also excellent in low-cost electric synapse, optical synapse and artificial synapse modulated by photoelectric combination [5,6,7,8,9,10,11]. CsPb (I, Br, Cl)_3_, as a representation of all-inorganic halide perovskites (IHPs), has been continuously considered, owing to its excellent physical properties, such as longer carrier diffusion length and higher carrier mobility [12]. Compared with other materials used in artificial neural synapses, such as oxides and two-dimensional materials, the preparation conditions of CsPbI_3_ are simpler and easier to synthesize [13,14,15,16,17,18,19,20]. In view of the excellent characteristics and neural morphology calculation of halide perovskites, it is necessary to further explore an artificial synaptic device with a simple preparation process and high performance. However, explorations of the application of IHPs to artificial synapses are remain rare.

Here, we report an artificial synapse based on CsPbI_3_ thin film with the structure of Au/CsPbI_3_/ITO. The synaptic plasticity in the Au/CsPbI_3_/ITO artificial synapse is realized, including long-term and short-term plasticity, paired pulse facilitation, paired pulse inhibition, spike time-dependent plasticity, and spike number-dependent plasticity, which signifies the biological synaptic function is successfully simulated, including the possibility of forming long-term memory. The properties of neural synapses are simulated with a relatively simple structure and low-cost synthesized method (see Table S1).

## 2. Materials and Methods

We adopted one-step spin-coating to fabricate the CsPbI_3_ thin film. We mixed CsI and PbI_2_ with 1:1 mole proportion and added them to 10 mL DMF using magnetic stirring for 30 min at a temperature of 30 °C. A golden clear solution was obtained.

After standing for one day, we observed that the solution had no precipitation and used a dropper to take an appropriate amount of solution and drop it evenly on an ITO glass, with spin-coating speed of 500 rpm for 15 s and 2000 rpm for 30 s. A pale yellow film was obtained after annealing at 100 °C for 5 min. The ITO glass was soaked in absolute ethanol and underwent ultrasonic cleaning for 15 min.

The sample was plated with a 0.2 mm diameter Au point electrode to form a top–bottom electrode structure. This operation was completed by a small vacuum coating machine and a mask with a diameter of 0.2 mm at room temperature. The thickness and the surface morphology of the CsPbI_3_ thin film was observed by field emission scanning electron microscopy (FESEM, Tescan, Brno, Czech Republic). Furthermore, the grazing-incidence X-ray diffraction (GIXRD, Bruker, Bremen, Germany) helped us complete the analysis of the phase structures of CsPbI_3_ films. The biological synaptic characteristics and current-voltage (*I*-*V*) were measured by a Keithley 2400 instrument (Solon, OH, USA).

## 3. Results and Discussion

### 3.1. Structure Analysis

**Figure 1:**
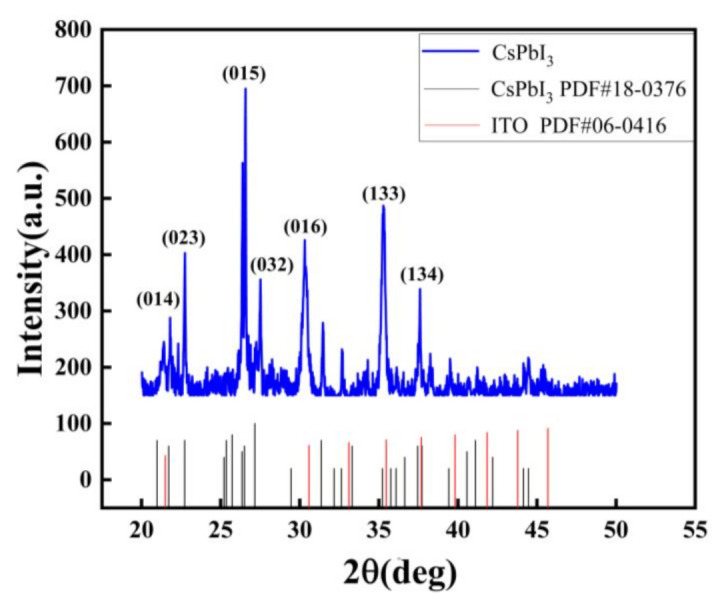
XRD patterns of the Au/CsPbI_3_/ITO artificial synapse.

Peaks from the δ-phase can be seen from the X-ray diffraction (XRD) pattern [21]. As shown in Figure 1, the crystal phases of diffraction peaks at 21.8 degrees, 22.7 degrees, 26.6 degrees, 27.5 degrees, 30.3 degrees, and 37.6 degrees are (014), (023), (015), (032), (016), and (134), respectively. However, the PDF card (No. 18-0376) of the CsPbI_3_ with yellow phase had diffraction peaks at 35.2 degrees, (133), perhaps indicating CsPbI_3_ tends to change to yellow phase in the air.

**Figure 2:**
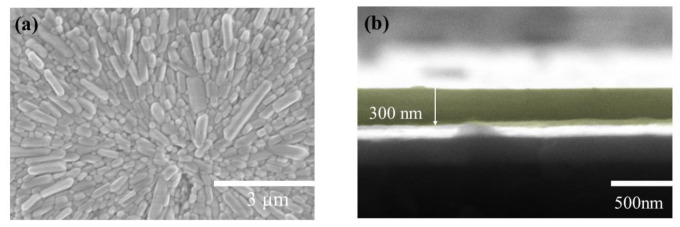
(**a**) The surficial SEM images of the Au/CsPbI_3_/ITO artificial synapse; (**b**)the cross-sectional SEM images of the Au/CsPbI_3_/ITO artificial synapse. The yellow part is CsPbI_3_.

**Figure 3:**
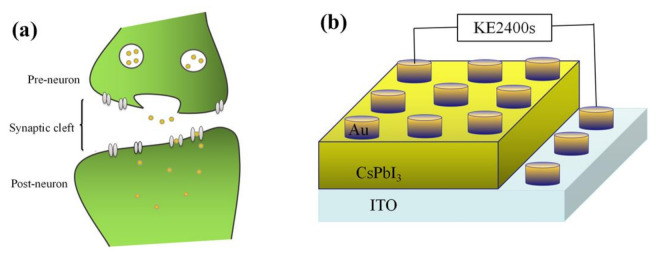
(**a**) A pair of synapses in biology; (**b**) the structure of the artificial synapse is Au/CsPbI_3_/ITO. The top electron is Au, and the bottom electron is ITO.

The surface of the artificial synapse can be observed in Figure 2a; few particularly obvious pinholes are on the surface of the CsPbI_3_ thin film, and the grains are relatively complete. As shown in Figure 2b, the thickness of the CsPbI_3_ thin film is approximately 300 nm. As shown in Figure 3a, a pair of synapses, the basis of information transmission in the central nervous system, include the pre-neuron, the post-neuron, and the synaptic cleft. Cells maintain a resting potential unless stimulated, and once stimulated, they will produce an action potential, which is called a bioelectrical phenomenon. Inter-synaptic signals are transmitted by neurotransmitters, by which the action potential reaches the front of synapses; neurotransmitters are released because of Ca^2+^ entering pre-synaptic neurons to promote the combination of synaptic vesicles and the plasma membrane. Then, neurotransmitters combine with post-synaptic cell membrane receptors to change the post-synaptic potential and complete the transfer of action potential [22].

Figure 3b shows that in the artificial synapse based on CsPbI_3_, the top electrode is regarded as the pre-neuron, while the bottom electrode is regarded as the post-neuron. The insulator is similar to a synaptic cleft. Electrical signals are applied to the top and bottom electrodes to simulate the release process of neurotransmitters.

### 3.2. Memory Behaviors

**Figure 4:**
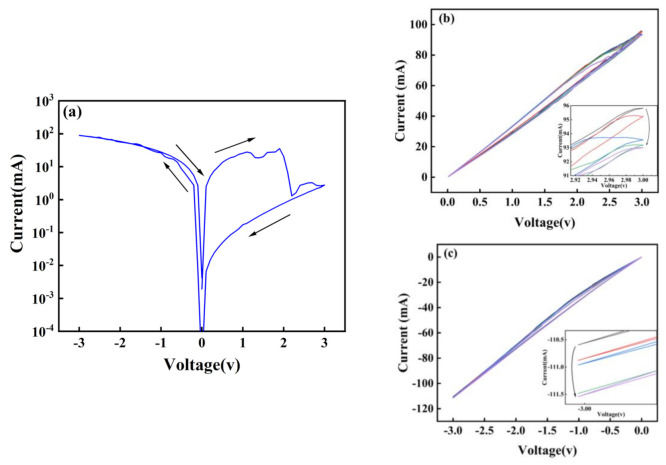
(**a**) The memory switching measurement of the Au/CsPbI_3_/ITO artificial synapse. The memory behavior of the Au/CsPbI_3_/ITO artificial synapse is measured by (**b**) five positive scanning voltage and (**c**) five negative scanning voltage.

Figure 4a shows the transition between high and low resistance states of the Au/CsPbI_3_/ITO artificial synapse. When a negative voltage is applied, the iodine ions migrate to the bottom electrode, the top electrode and the bottom electrode are connected by the iodine vacancy conductive filaments, and the opposite voltage drives the filaments to decompose. Therefore, the device can switch between high and low resistance states. The formation and decomposition of conductive filaments make it possible for pulse modulation (voltage, spike-number, frequency) to induce the synaptic behavior of devices [23,24,25,26,27,28]. Figure 4b,c illustrate that the artificial synapse has learning and memory behavior similar to neural synapses. With a variety of stimuli, experience-dependent changes in synaptic weight occur, leading to the remodeling of neural circuits, which is called synaptic plasticity. The synaptic plasticity turns experience to memory, divided into short-term memory and long-term memory [29]. Controllable synaptic weight changes indicate that the device has basic learning and memory behaviors similar to synapses.

### 3.3. The Synaptic Properties

**Figure 5:**
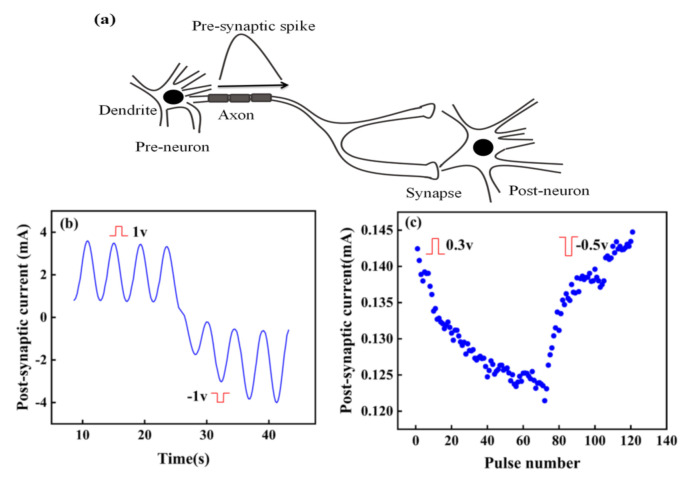
(**a**) Signal transmission between two neurons; (**b**) Response of current to time under continuous positive and negative pulses. (**c**) Potentiation and depression of current.

In biology, synapses can be excitatory or inhibitory, specifically manifested as the increase or decrease of post-synaptic current (PSC). Figure 5a shows the signal transmission between two neurons. When the pre-spike arrives at the post-neuron, the state of post-neuron will change and be in a state of excitation or inhibition. Excitation and inhibition in a synapse depend on the change of the probability of action potential in post-synaptic cells, with the increase of action potential of pre-synaptic neurons [30]. Similarly, the conductance of the artificial synapse, a quantitative representation of the synaptic weight, can increase or decrease with the input voltage pulses.

The artificial synapse has the characteristics of potential potentiation and depression by changing the polarity of input pulse. As shown in Figure 5b, stimulated by continuous positive voltage, the PSC of the artificial synapse decreases, while the PSC increases under continuous negative voltage. Figure 5c further reveals this phenomenon. When positive pulses are applied to the device, the PSC continues to decrease, indicating the synaptic device is in a state of excitation. However, the PSC continues to increase when pulses turn to negative ones, which demonstrates the artificial synapse is in the inhibition state. Excitation and inhibition can change the synaptic weight of the artificial synapse, further illustrating the synaptic plasticity in the artificial synapse, which can possibly contribute to memory formation.

### 3.4. The Synaptic Plasticity

Two manifestations are mentioned in the synaptic plasticity: one is short-term synaptic plasticity (STSP), and the other is long-term synaptic plasticity (LTSP). As the capacity of a pre-synaptic input to influence post-synaptic output, the effects of synaptic weight can be either enhancement or depression. In the STSP, enhancement and depression in synaptic weight only last for a short time. Compared to the STSP, in the LTSP, enhancement and depression can last for a longer time [31].

**Figure 6:**
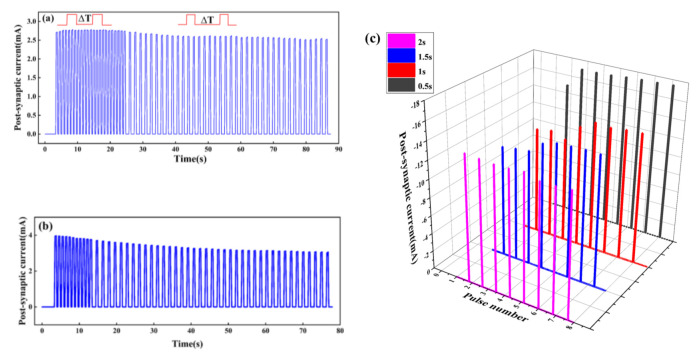
Response of current to voltage under two pulses with different time intervals (Δ*T*). The voltage of pulses is 0.5 v: (**a**) Δ*T* = 0.3 s, lasting for 20 s, and Δ*T* = 1 s, lasting for 60 s; (**b**) Δ*T* = 0.3 s, lasting for 10 s, and Δ*T* = 1 s, lasting for 60 s; (**c**) four sets of pulses with different Δ*T* are applied to the Au/CsPbI_3_/ITO artificial synapse. The value of Δ*T* is 0.5, 1, 1.5, and 2 s, and the amplitude is –0.5 V.

There are four states of potentiation and depression of the synaptic weight: short-term potentiation (STP), long-term potentiation (LTP), short-term depression (STD), and long-term depression (LTD). STSP includes STP and STD, while LTP and LTD are classified as LSTP. In the STP state, the synaptic weight rises temporally and then decreases rapidly to its original state. When repeated input pulses are applied, a permanent change occurs, suggesting the synaptic weight is in the LTP state. The STD state means the temporal decay of synaptic strength, but a permanent low value of synaptic weight represents the LTD state [32]. Shown in Figure 6a, the PSC increases after a long period of high-frequency stimulation, indicating the synaptic weight is enhanced. When Δ*T* = 0.3 s lasts for 20 s, the PSC first increases and then tends to be flat, which indicates that repeated stimulation will stabilize the enhancement of synaptic weight and help to form long-term memory, while when Δ*T* = 1 s lasts for 20 s, the PSC decreases, which indicates the artificial synapse is in a state of inhibition. However, Figure 6b shows that the short-term high-frequency stimulation will not enhance synaptic weight, but rather puts the synaptic weight in a LTD state.

When Δ*T* = 0.3 s and lasts for 10 s, the PSC decreases, and the artificial synapse is inhibited. When Δ*T* = 1 s, the PSC further decreases and finally tends to be flat, and the synaptic weight is in a LTD state.

The response of current to pulses at different Δ*T* is interesting. As shown in Figure 6c, four sets of pulses with different Δ*T* are applied to the Au/CsPbI_3_/ITO synaptic device. When Δ*T* = 2 s, the current decreases and the synaptic weight is inhibited, while when Δ*T* = 0.5, 1, and 1.5 s, the current increases, indicating that synaptic weight is enhanced. In addition, compared to Δ*T* = 1 s and Δ*T* = 1.5 s, the current increases faster to the maximum and lasts longer when Δ*T* = 0.5 s. It seems that pulses at a shorter time interval are conducive to the stable change of synaptic weight and the formation of long-term memory.

### 3.5. The Spike-Number-Dependent of Au/CsPbI_3_/ITO Artificial Synapse

**Figure 7:**
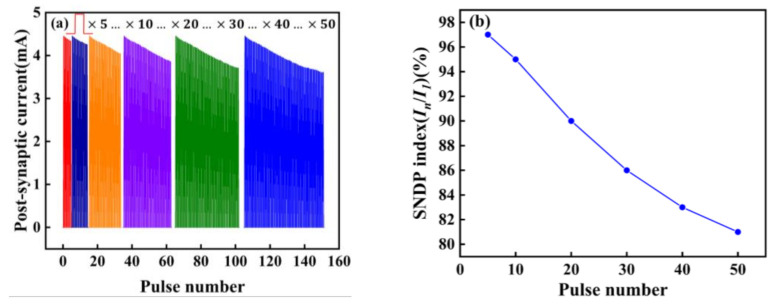
(**a**) SNDP; (**b**) SNDP index (*I_n_*/*I_1_*) of the Au/CsPbI_3_/ITO synaptic artificial synapse. The number of pulses is 5,10, 20, 30, 40, and 50. (*I_n_*/*I_1_*) is the ratio of the amplitude of the PSC under the last pulse stimulation to the amplitude of PSC under the first pulse stimulation. The voltage of pulses is 0.5 V.

We also explored the spike-number-dependent (SNDP) of the artificial synapse, which is one of the synaptic properties [9]. It can be seen from Figure 7a,b, that when five different numbers of pulses are applied to the device, the SNDP index (In/I1) is obtained. With the increase of the number of pulses, the PSC gradually decreases, indicating artificial neurons are gradually inhibited. When fifty pulses are applied, the SNDP index reaches 80%, and artificial neurons are inhibited to the greatest extent compared to the first five pulses.

### 3.6. The Paired-Pulse Facilitation and Paired-Pulse Depression

As typical behaviors of STP and STD, the paired-pulse facilitation (PPF) and paired-pulse depression (PPD) are pivotal for both excitatory and inhibitory responses between adjacent synaptic connections [33]. Two pulses are applied to the Au/CsPbI_3_/ITO artificial synapse. Compared to stimulation by the first pulse, the PSC increases after the second pulse if the device is in PPF state. The increase of post-synaptic current is inversely proportional to the time interval between two pulses. PPF and PPD are important in decoding the temporal information of visual and auditory signals, which reflects the emergence of recent spikes, so as to convert the temporal information into spatial data. In visual systems, via a pre-synaptic train of action potentials, PPF has dynamically controlled properties. It can endow different geniculate retina synapses, which makes synapses respond more abundantly on a time scale [34].

**Figure 8:**
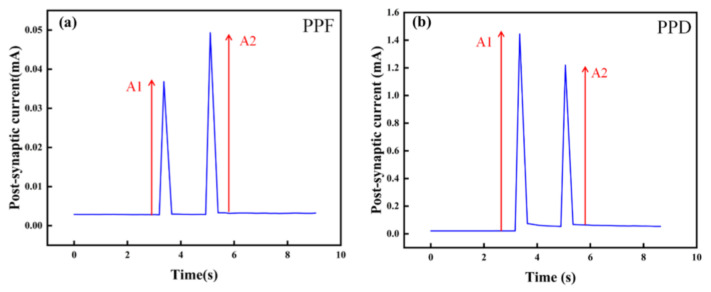
STSP of the Au/CsPbI_3_/ITO artificial synapse. The amplitude of pulses is 1 V. A1 is the amplitude of the PSC after the first pulse. A2 is the amplitude of the PSC after the last pulse. (**a**) PPF state in the Au/CsPbI_3_/ITO artificial synapse. (**b**) PPD state in the Au/CsPbI_3_/ITO artificial synapse.

In order to study the STSP of the artificial synapse, we designed PPF and PPD experiments. As shown in Figure 8a,b, PPF and PPD were observed when a pair of pulses with an amplitude of 1 V were applied to the artificial synapse. In the PPF state, the PSC of the device increases after the last pulse, while in the PPD state, the PSC decreases. PPF and PPD illustrate the STSP in the artificial synapse, indicating the possibility of short-term memory formation.

### 3.7. The Spike-Timing-Dependent Plasticity

Synapses play an important role in the transmission of signals between neurons and the memory and learning behaviors of the brain. Spike-timing-dependent plasticity (STDP), also called Hebbian learning, shows the fundamental function of synaptic plasticity in a biological synapse.

In the Hebb hypothesis, continuous and repeated stimulation will induce changes in the cells, such as some growth processes and metabolic changes. In the STDP state, pre-synaptic neurons continuously stimulate post-synaptic neurons, so the connection degree between neurons changes, which is an embodiment of LTSP. The concept explains cellular learning in the neural computing system. The asymmetric properties of STDP were found in cultures of hippocampal cells. The degree and direction of modification of synaptic weights is determined by the relative timing of pre-synaptic spikes and post-synaptic spikes. When a pre-synaptic spike arrives at time *t_pre_* before or after the post-synaptic spike time *t_post_*, the synaptic weight changes, and LTP or LTD behavior is induced [35].

**Figure 9:**
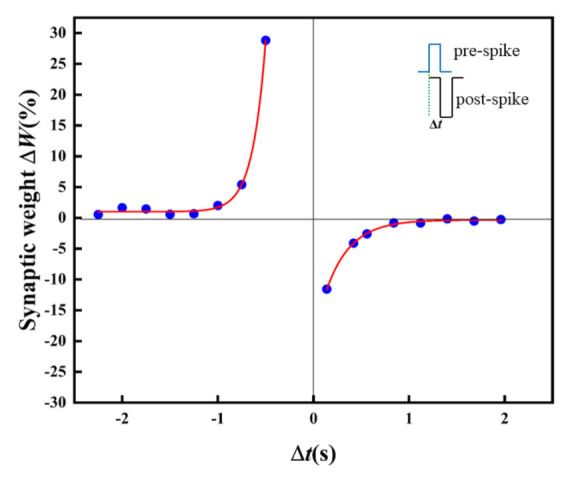
STDP of the Au/CsPbI_3_/ITO artificial synapse. The relative change of the synaptic weight (Δ*W*) versus the relative spike timing (Δ*t*); Δ*t*=*t_post_* − *t_pre_*. The blue dots are the measured data, and the red solid line is the result of fitting by the Origin 2021.

In biology, the STDP learning rule is a typical LTSP. As one of the basic learning rules for simulating synaptic functions, the synaptic weight can be enhanced/inhibited by adjusting the spike time or sequence through STDP [36]. For the sake of the STDP function realization, pulses with an amplitude of 0.3 V and −0.5V are imposed on the artificial synapse as the pre-synaptic spikes and post-synaptic spikes. Figure 9 displays the STDP in the artificial synapse. The relative change of synaptic weight (*ΔW*) changes with the change of relative time (Δ*t*). The artificial synapse is in the LTP state is at Δ*t* < 0. However, the artificial synapse turns to LTD at Δ*t* > 0.

In the case of pre-synaptic spikes or post-synaptic spikes stimulated earlier than post-synaptic spikes or pre-synaptic spikes, long-term potentiation or inhibition is observed, and the absolute value of Δ*W* increases exponentially with decreasing Δ*t*. The STDP in biological synapses is expressed as follows, including the Symmetric Hebbian and antisymmetric Hebbian learning rules [37].

Δ*W*= *A* exp(−Δ*t*^2^/*τ*^2^) +Δ*W*_0_ (1)

Δ*W = A* exp*(−Δt/τ) +*Δ*W_0_* (2)

*A* and *τ* are the scaling factor and time constant, respectively, and Δ*W* is the relative change of the synaptic weight (Δ*W* = (*W*_post_ − *W*_pre_)/*W*_pre_). The experimental data of Δ*W* and *τ* could be well fitted with the exponential decay function (Equation (2)) in the antisymmetric Hebbian learning rules, where *A*_1_ is −19.4, *τ_1_* is 0.26(Δ*t* > 0), *A*_2_ is 1078.7, and *τ_2_* is –0.14 (Δ*t* < 0).

## 4. Conclusions

We designed an artificial synapse based on halide perovskite. We successfully fabricated the CsPbI3 thin film and adopted a structure of Au/CsPbI_3_/ITO. The Au/CsPbI_3_/ITO artificial synapse exhibited depression/potentiation after positive/negative pulses, which indicated a performance similar to that of a neural synapse in biology. In addition, the Au/CsPbI_3_/ITO artificial synapse exhibited favorable synaptic plasticity, including STSP and LTSP. STSP is divided into STP and STD. The device displayed a typical behavior of STP (PPF) and another typical behavior of STD (PPD). The phenomenon of STDP indicates LTP and LTD in the device, which is a typical behavior of LTSP. We explored the possibility of long-term memory formation through applying pulses with different time intervals and different pulse durations. The results indicate that the high-density durable stimulation is conducive to long-term memory formation. As one of the synaptic properties, the device also reflects the SNDP and the SNDP index, showing that the number of pulses causes a change of the PSC.

## Data Availability

The data that support the funding of this study are available from the corresponding author upon reasonable request.

## Supplementary Materials

The following supporting information can be downloaded at: https://www.mdpi.com/article/10.3390/mi13020284/s1, Table S1: Structure, Synthesized method and Synaptic properties of artificial neural synapses based on oxide and 2D materials.
