# Supplementary material for: An Artificial Synapse Based on CsPbI3 Thin Film
Source: Micromachines (Basel). 2022 Feb 10;13(2):284. doi: 10.3390/mi13020284 (PMC8874570; doi:10.3390/mi13020284)
Supplement: Supplementary file 1 [file micromachines-13-00284-s001.zip › micromachines-1559196-supplementary.pdf]

Article

An artificial synapse based on CsPbI3 thin film

Jia-Ying Chen, Xin-Gui Tang*, Qiu-Xiang Liu, Yan-Ping Jiang, Wen-Min Zhong and Fang Luo

Table S1. Structure, Synthesized method and Synaptic properties of artificial neural synapses based on oxide and 2D materials.

Materials	Structure	Synthesized method	Synaptic properties	References
TaOx HfO_2	Pt/TaOx/HfO_2/ TiN	Atomic layer deposition (ALD)	Potention and Depression	[1]
TiO_2 SiO_2	TiN/Ti/TiO_2/ SiOx/Si	DC sputter low-pressure chemical vapor deposition (LPCVD)	PPF STDP STP	[2]
Al_2O_3	Pt/Al_2O_3/TiN	DC sputter ALD	PPF LTP STP	[3]
Al_2O_3 HfO_2	TaN/HfO_2/Al_2O_3/HfO_2/ITO	ALD	PPF PPD STDP	[4]
Nb_2O_5	two-dimensional architecture consisting of a $NbSe_2$/WSe_2/ Nb_2O_5 heterostructure	Chemical evaporation chemical vapor deposition (CVD)	post-synaptic current modulation	[5]

References

1. Ryu, H.; Kim, S., Pseudo-Interface Switching of a Two-Terminal TaOx/HfO_2 Synaptic Device for Neuromorphic Applications. *Nanomaterials (Basel)* **2020**, 10 (8) , 1550.
2. Cho, H.; Kim, S., Short-Term Memory Dynamics of TiN/Ti/TiO_2/SiO_x/Si Resistive Random Access Memory. *Nanomaterials (Basel).* **2020**, 10 (9) ,1821.
3. Ryu, H.; Kim, S., Synaptic Characteristics from Homogeneous Resistive Switching in Pt/Al_2O_3/TiN Stack. *Nanomaterials (Basel).* **2020**, 10 (10) , 2055 .
4. Mahata, C.; Kang, M.; Kim, S., Multi-Level Analog Resistive Switching Characteristics in Tri-Layer HfO_2/Al_2O_3/HfO_2 Based Memristor on ITO Electrode. *Nanomaterials (Basel).* **2020**, 10 (10) ,2069 .
5. Park, W.; Jang, H. Y.; Nam, J. H.; Kwon, J.-D.; Cho, B.; Kim, Y., Artificial 2D van der Waals Synapse Devices via Interfacial Engineering for Neuromorphic Systems. *Nanomaterials.* **2020**, 10 (1) ,88 .
